# Xerostomia as an oral-systemic indicator: Etiology, diagnostic strategies and multimodal management approaches

**DOI:** 10.6026/973206300221118

**Published:** 2026-02-28

**Authors:** Anisha Parani, Anuja Nimbalkar, Harshbala Sharma, Alona Joppan, Anish Raj Godavarthy, Walid Ourdane

**Affiliations:** 1 Department of Dental Surgery, KGF College of Dental Sciences, Karnataka, India; 2 Department of Dental Surgery, Maharashtra University of Health Sciences, Nashik, Maharashtra, India; 3 Department of Dental Surgery, Saraswati Dental College, Lucknow, Uttar Pradesh, India; 4 Department of Dental Surgery, Kerala University of Health Sciences, Thrissur, Kerala, India; 5 Department of Dental Surgery, Manipal College of Dental Sciences Manipal, Karnataka, India; 6 Department of Dental Surgery, Doctor of Dental Medicine, Dental Department, Faculty of Medicine, Algiers University, Algiers, Algeria

**Keywords:** Xerostomia, disease-induced xerostomia, medication-induced xerostomia oral health complications, management strategies

## Abstract

Xerostomia of the oral cavity reflects multifaceted problems in salivary gland functions with implications for oral and systemic health. The various etiological elements range from disease-related glandular damage, therapeutic interventions and an extensive spectrum of medications with xerogenic potential. Complications such as caries incidence, periodontal issues and prosthetic instability emphasize timely clinical assessment and targeted management. This review synthesizes current evidence on etiology, disease and medication- induced xerostomia. It is also associated with oral health challenges, assessment strategies and preventive education. Awareness, understanding and addressing xerostomia comprehensively can mitigate morbidity, preserve oral function and enhance quality of life.

## Background:

The term xerostomia, which translates to "dry mouth," comes from the Greek word stomia (mouth) and Xero (dry) and refers to a persistent feeling of oral dryness caused by diminished salivary secretion (hyposalivation) [1]. It may or may not be associated with hyposalivation and can occur due to abnormal salivary gland function known as true xerostomia; however, some patients experience a feeling of dryness in the mouth despite normal salivary flow, classified as pseudo or symptomatic xerostomia [1]. This is a clinical symptom and not a disease, but often when present, signals towards a deeper root cause like glandular malfunction, radiotherapy, pharmacological and underlying systemic issues, as highlighted in recent work on salivary physiology and flow regulation [2]. Saliva is a cardinal biological fluid that has a significant role in maintaining oral lubrication, digestion, chewing, swallowing, PH buffer, preserving homeostasis and antimicrobial activity, but when secretion is impaired oral health and quality of life in general are compromised [3]. Therefore, it is of interest to report the etiological factors, clinical significance and multidisciplinary approach for clinical management, preventive measures and patient education to alleviate the manifestations.

## Xerostomia:

## Overview, etiology and clinical significance:

## Etiology:

The reasons behind xerostomia are multifactorial and diverse. However, dry mouth is not only an age- associated consequence; other causes that change the salivary flow rate and its composition are also drug-induced, chemotherapy, radiation- induced systemic disease, autoimmune, infection-related, psychological disease and glandular pathologies related to xerostomia. Among all the etiologies, medication-induced xerostomia is most common and it is frequently seen in patients on multiple drug prescriptions. Most prescribed medications causing xerostomia are antidepressants, diuretics, antihypertensives, SSRIs, anticholinergics and sedative drugs. Polypharmacy in old age patients and comorbidity compound the xerogenic effect and make it worse. Another etiological factor which accounts for xerostomia is disease-induced xerostomia, which includes systemic disease (metabolic or autoimmune diseases. Patients suffering from head and neck cancer always go through Chemotherapy /radiation during the treatment process and these treatment modalities also induce injury, which impairs the salivary gland function [4]. Aging, lifestyle, glandular pathologies, degenerative changes in salivary-gland tissue and salivary gland tumor changes in ductal obstruction, acinar atrophy and sialolithiasis. Psychological stress, anxiety, depression and neurological disorders like Parkinson's, Alzheimer's and injury to the facial nerve impair the parasympathetic activity, resulting in reduced saliva release [5].

## Clinical significance:

It is true that the importance of saliva becomes most evident when its production is compromised. However, clinically, there are more severe consequences of xerostomia than just discomfort; patients find it difficult to perform oromotor functions such as speaking, swallowing and chewing [3]. In addition to this, prosthetic and geriatric patients suffer with compromised denture retention due toa lack of natural lubrication and muco-adhesion, leading to frictional irritation and discomfort in wearing [6]. Among many, one of the clinical significances of xerostomia is insufficient physical cleaning, buffer acid PH imbalance and change in oral microbiome resulting in oral dysbiosis, halitosis, ulceration, burning sensation and growth of opportunistic infections such as Candida albicans, elevated caries risk (particularly for root and cervical lesions), gingival and periodontal disease, plaque accumulation [7]. Moreover, with altered rheological demand of the oral cavity, the composition gets altered, causing imbalance in electrolyte ions (dehydration), minerals, mucin, peptides, digestive enzymes, lysozymes, immunoglobulin, growth affecting antimicrobial function, nutritional impairment, delayed wound healing by disrupting the repair and regeneration mechanism and lower immunity level [7]. As the protective function of saliva is damaged this atrophy of papilla is known as atrophic glossitis, translating into dysgeusia. Physiologically and pathologically, saliva is an excellent source of biomarkers for disease detection [8]. Ultimately, xerostomia has a substantial impact on oral and general health and is a systemic indicator of physiological imbalance rather than merely a localized oral complaint.

## Disease-induced xerostomia:

Among the various factors contributing to salivary gland dysfunction, systemic diseases act as the main cause, profoundly impacting both glandular structure and functional capacity. This will result in a decrease in salivary flow which is not a side effect but a direct effect of autoimmune, metabolic or neurologic diseases that fundamentally affect the normal functioning of the salivary gland. Understanding the intricacies of these underlying conditions is not just beneficial, but truly essential for immediate diagnosis and for the necessary multidisciplinary treatment plan. A crucial factor for maintaining the delicate microbial balance, lubrication and pH stability within the oral cavity is saliva and a sustained decrease in its flow can lead to a host of complications, including fungal infections, aggressive dental caries and persistent mucosal discomfort. It is critical to note that while drug-related etiologies are well publicized; numerous systemic diseases directly and often quite severely impact both the quantity and composition of these glandular secretions.

## Autoimmune and inflammatory etiologies:

Some of the causes that have been extensively studied and documented by clinical researchers are the autoimmune diseases and SjÖgren's syndrome, for example, being the classic case. In this case, the gradual acinar tissue destruction is mediated by lymphocytic infiltration, a process that ultimately results in severe, characteristic dryness also known as Xerostomia and, frequently, noticeable parotid gland enlargement. Other related autoimmune pathologies such as Systemic Lupus Erythematous, Rheumatoid Arthritis worsening the salivary gland dysfunction [9].

## Endocrine and metabolic disorders:

Among the other major causes contributing to salivary gland dysfunction are a range of endocrine and metabolic disorders, the most prominent among them being Diabetes Mellitus. Diabetes compromises the critical physiological components, which include all the microvascular circulation, autonomic nervous system control and the electrolyte balance within the salivary glands themselves. During periods of uncontrolled hyperglycemia and general dehydration, exacerbation of these symptoms is usually observed and this makes the management of blood glucose levels very important. Other related endocrine conditions, such as adrenal insufficiency and various forms of thyroid dysfunction, can also measurably alter systemic water balance and glandular metabolism, resulting in dryness, the cause of which can be clinically confirmed [10].

## Renal and hepatic dysfunction:

Renal and hepatic diseases cause dry mouth due to a mix of systemic issues, especially uremic toxicity, limited fluid intake and changes in salivary composition. In conditions of chronic kidney disease, high levels of urea in the blood significantly affect both taste and the important buffering ability of saliva. However, a person with liver cirrhosis, the main issue is a decrease in plasma protein production. This can disrupt the body's fluid balance, which largely contributes to the overall sense of dryness [11].

## Infectious, neurologic and other systemic causes:

Etiologies like infectious diseases further worsen the clinical condition, for instance, HIV infection directly invades the glandular epithelium, resulting in the formation of lymphoepithelial lesions and noticeable cystic enlargements. Other infectious causes like Hepatitis C trigger an autoimmune sialadenitis that closely mimics the presentation of SjÖgren's syndrome, while granulomatous disorders like sarcoidosis and tuberculosis are known to physically infiltrate the salivary tissue and thus affect its function [12]. Furthermore, we must also consider neurological and mental health conditions such as Parkinson's and Alzheimer's disease, which can dramatically disrupt the central autonomic regulation of saliva and common depression and anxiety, which can indirectly affect the sympathetic nervous system and the neurotransmitters of the body whereby resulting in the sensation of persistent dry mouth. Finally, we must also consider the effects of hematological and oncological diseases, such as lymphoma, leukemia and graft-versus-host diseases (which typically occur after transplantation), on the sensation of dryness in the oral cavity. These conditions can cause direct or immune related damage to the salivary glands. While conditions like chronic malnutrition or cystic fibrosis typically lead to dehydration and blocked salivary ducts [13].

## Diagnosis and management:

Accurate diagnosis of this condition requires understanding of the patient's reported symptoms and their systemic indicators and for this, specialized salivary flow tests also known as sialometry and in specific cases, glandular biopsies or comprehensive blood tests are performed. Xerostomia usually occurs in a person before a systemic disease is identified and for this reason, usually dentists bear the initial responsibility of suspecting and initiating an evaluation for an underlying medical issue. Treatments for this condition generally involve a combination of systemic disease management and targeted localized therapies. Simple behavioral modifications like encouraging patients to stay well hydrated, advising them to rigorously avoid alcohol and caffeine and suggesting the use of sugar-free chewing gum or lozenges can help stimulate saliva production. For those patients whose salivary glands still have functional capacity, effective pharmacologic options like pilocarpine or cevimeline can be used to promote salivary secretion. The standard localized regimen includes topical fluoride application, antimicrobial mouthwashes and routine dental check-ups. There are also emerging biological and regenerative therapies under development for the goal of restoring the lost salivary gland function. Disease-induced xerostomia provides a glimpse into a patient's complete systemic health and needs comprehensive work-up for possible conditions. To avoid issues and enhance life of the patient, timely detection and management would be needed. As some medications can also affect the salivary glands, distinguishing disease-related causes from drug-induced xerostomia is essential.

## Medication-Induced xerostomia:

Dry mouth due to medicines is quite common in dental practice. Many times it is not noticed until patients complain. This dryness is different from cases caused by diseases like diabetes or SjÖgren's syndrome. Here, the problem comes because certain medicines disturb the normal work of salivary glands. It is mostly seen in older people and those who take medicines for a long time. So, dentists should keep this in mind while treating such patients. Saliva is very important for oral health. It helps in chewing, swallowing and digestion. It also protects the mouth from germs. Saliva keeps teeth strong by balancing minerals. When medicines reduce saliva, patients may feel dryness, have trouble speaking or eating and may get more cavities or infections. These problems can also affect dentures and other dental work. What looks like a small issue can slowly become serious if not managed. Many medicines are known to cause dry mouth. They may block nerve signals, reduce body fluids or affect the brain. Wolff and colleagues found that more than 50 types of medicines can affect the salivary glands [14]. This shows why it is important to check the patient's medicine list during dental visits.

Common medicines that can cause dry mouth;

[1] Tricyclic antidepressants - e.g., amitriptyline

[2] Selective serotonin reuptake inhibitors (SSRIs) - e.g., fluoxetine

[3] Beta-blockers - e.g., propranolol

[4] Calcium channel blockers - e.g., amlodipine

[5] Antimuscarinic agents - e.g., oxybutynin

[6] Diuretics - e.g., hydrochlorothiazide

[7] Antihistamines - e.g., diphenhydramine

[8] Benzodiazepines - e.g., diazepam

All of these medicines affect saliva in different ways, but the result is dryness. Some act directly on the glands, while others reduce fluid in the body or slow down the nervous system. Even medicines with mild effects can cause problems when used for a long time or together with other drugs. Older people are more affected because they often take many medicines and their glands may not work properly due to age [15]. In old age homes or care centers, dry mouth is often not reported, which delays treatment and increases oral problems [16]. Dentists should ask patients directly, as many may not mention it unless questioned. To understand dry mouth, it helps to know how saliva works. Recent studies on salivary ion composition and flow regulation show that saliva plays a key role in protecting the mouth and supports in healing [2]. Even a small drop in saliva can disturb this balance and cause symptoms. Measuring saliva is not always easy, especially when patients feel dry but still produce some saliva. Unstimulated saliva, collected without chewing, gives a better idea of how the glands are working. Recent studies show that dry mouth from medicines can also increase pain in the mouth or face. Kohli and team found that it may make nerve or muscle pain worse [17]. Simple steps like using saliva substitutes, drinking more water or chewing sugar-free gum can help. Oil pulling with coconut oil has also been reported to reduce symptoms, giving patients a non-drug option for relief. In dental clinics, it is important to ask patients if their medicines are causing dry mouth. Dentists can consult with doctors to adjust or change medicines when possible. A good plan includes fluoride treatment, saliva stimulants and regular check-ups to keep the mouth healthy. Patient education is also important, as many people may not realize that their medicines are linked to oral problems. To summarize, dry mouth caused by medications is a significant issue in dental care. With proper checking, patient education and preventive steps, dentists can reduce the problems and help patients keep their mouth healthy. As more people use long-term medicines, this should become a regular part of dental care. In this context, the following section mainly discusses the impact of xerostomia on the progression of caries, periodontal stability and prosthetic management.

## Challenges:

## Caries, periodontal disease and prosthetic complications in xerostomia:

Xerostomia converts the self-regulating oral cavity into an environment more susceptible to microbial growth. The hard tissues, soft tissues and prosthetic interfaces each experience distinct challenges due to decreased salivary flow and quality. Saliva usually has protective and re-mineralizing properties which maintain neutral pH while delivering essential ions like calcium and phosphate. Thus, reduction or compositional change in the salivary flow enhances bacterial adhesion and acidogenic activity which leads to rampant coronal and root-surface caries [18]. This also promotes marginal microleakage, seal failure and secondary caries. The life of resin composites and glass ionomers cements is shortened when subjected to xerostomic conditions due to dehydration and microfracture risk. Research suggests that a direct link between xerostomia and periodontal disease is quite complex. However, the reduction of salivary protective shield undeniably affects periodontal stability. Inadequate saliva diminishes cleansing and lubrication function, which in turn fosters plaque stagnation and gingival inflammation [4]. Mori *et al.* reported that patients with systemic-disease-associated-xerostomia often exhibit lower oral pH. This provides a conductive environment for fungal colonization which triggers periodontal issues like gingivitis, candidiasis and delayed wound healing. Deficiency of antimicrobial salivary peptides such as lysozyme and lactoferrin often disrupts the subgingival microbial balance, which fosters the growth of anaerobes like porphyromonas gingivalis and fusobacterium nucleatum. The resulting inflammation has the potential to aggravate attachment loss and hinder post-operative healing. Re-establishing occlusal function in addition with improved hydration and managing systemic health has collectively shown to enhance the periodontal outcomes in xerostomic patients [19]. Saliva is highly regarded to facilitate denture retention through its physical properties such as adhesion, cohesion and surface tension which ensures smooth movement and mucosal comfort. When these mechanisms fail, patients will experience pain, ulceration and frequent dislodgement. Lysik *et al.* found that more than 70% of denture wearers with xerostomia reported soreness when compared with only 15% of non-xerostomic users. Thus, among other oral challenges, prosthetic management in xerostomic patients poses the greatest functional limitations. Conventional saliva substitutes and moisturizing gels only offer partial relief as their physical properties are starkly different from natural saliva. It is established that most commercial gels are more viscous and elastic which limits their ability to spread easily and form a stable film. Emerging saliva-substitute technologies increasingly aim to replicate natural saliva's rheological properties, including viscosity, elasticity and film-forming behavior [18]. Denture base materials such as polymethyl methacrylate also exhibit poor wettability, increasing friction and microbial adhesion, which predisposes to denture stomatitis [20]. Newer formulations explore mucoadhesive and antimicrobial approaches to improve long-term comfort and overall xerostomia management [21]. Since xerostomia disrupts the oral ecosystem's physicochemical balance, it could thus be attributed to significantly amplifying the risk for dental caries, periodontal deterioration and prosthetic failure. Successful management extends beyond symptom control as it requires preventive, material-based and interdisciplinary approaches that compensate for saliva's protective functions. The subsequent section therefore discusses the detailed diagnosis and management strategies of xerostomia.

## Clinical assessment and management strategies of xerostomia:

## Clinical assessment:

## Patient history and symptom inquiry:

A medical history with a focus helps determine xerostomia. Ask about the duration, the start time, the changes during the day, trouble swallowing, changes in taste, trouble speaking, discomfort from dentures and burning mouth syndrome. A full medication review is indicated since numerous common medications in the form of antidepressants, antihypertensives, anticholinergics, antihistamines and opiates possibly lead to or exacerbate dry mouth [22]. Consider systemic diseases like Sjögren's syndrome, diabetes, past radiation therapy to the head and neck and harmful social habits like tobacco use, alcohol use and caffeine ingestion because they cause problems.

## Standardized symptom questionnaires:

Standardized questionnaires for assessment of the symptom burden are available for baseline and follow-up assessments, such as the Xerostomia Inventory (XI) and the short form (SXI). The full XI is reproducible and has had its range and minimal important difference documented recently in the literature, making it a useful instrument for comparisons and assessing response to treatment [23].

## Oral clinical examination:

An intraoral examination should focus on mucosa surface wetness, area of erythema and atrophy, tongue fissures, caries, plaque/ periodontal status, denture fit, decreased pooling of saliva in the floor of mouth and increased mucosal friability. Oral microbiome shifts and candidal overgrowth due to hyposalivation are often seen in cases of secondary infection [20]

## Objective salivary measurement:

Objective methods supplement these reports, including the use of unstimulated whole saliva (UWS) and stimulated saliva flow rates (SFR). Currently, researchers define hyposalivation with UWS cutoffs of 0.1-0.2 mL/min and for stimulated (SWS) cutoffs of <0.5-0.7 mL/min [24]. Saliva collection duration and timing can vary across studies. It is therefore critical that standard collection methods (e.g., 5-minute saliva collection for unstimulated samples) and other details are provided in the reporting.

## Adjunctive diagnostics:

If SGH is accompanied by a strong suspicion for systemic disease such as Sjogren's disease, further investigation may include serological tests for anti-SSA/SSB antibodies, sialography/scintigraphy, ultrasound/MRI of the salivary glands or a salivary gland biopsy. In suspected radiation-induced SGH, staging can be performed by using imaging and reviewing oncology records [24].

## Management strategies:

Management of xerostomia should be individualized and multi-modal, combining (1) addressing reversible causes, (2) symptomatic measures, (3) salivary stimulation when gland function remains and (4) preventive oral care to reduce complications.

## Address reversible and contributing factors:

First-line management may include medication review and change (substituting non-anticholinergic medication and adjusting doses), optimizing control of co-morbidities that reduce salivation [22] and general recommendations such as drinking more water and reducing tobacco, alcohol and caffeine consumption if their use is shown to contribute to symptoms.

## Symptomatic, behavioral and topical measures:

Polypharmacy is avoided. Small, frequent amounts of water, sugar-free chewing gum and xylitol lozenges (which both increase saliva and reduce caries) may be helpful. Sleeping in a humidified atmosphere may help. Over-the-counter carboxymethylcellulose products or mucins in spray, gel or lozenge temporarily improve symptoms and are generally recommended. The quality of various saliva substitutes has been assessed and some carboxymethylcellulose-based substitutes have good wetting/lubricating properties. Patients should be educated on the appropriate use and the frequency of testing [25].

## Pharmacologic salivary stimulants:

For those with some residual gland function, systemic parasympathomimetics (including pilocarpine and cevimeline) are the most strongly evidence-supported for improving subjective symptoms and objectively measured flow. Pilocarpine 5 mg three times per day and cevimeline 30 mg three times per day (where available). Although they increase flow, they are cholinergic (can cause sweating, stomach upset, frequent urination) and require close medical supervision [25]. ASCO and other specialty guidelines recommend them for carefully selected persons after radiation failure only if the possible side effects are outweighed by other factors [22]. Topical stimulants such as citric acid sprays or malic acid lozenges may temporarily stimulate the salivary glands, but caution is advised due to their erosive potential.

## Preventive and dental-focused strategies:

Because xerostomia increases the risk of caries and periodontal disease and may weaken prosthodontics, dental preventive measures are recommended, such as: 1.1% sodium fluoride toothpaste, fluoride varnish or trays, as well as follow-up professional hygiene recall appointments and antimicrobial mouth rinses for patients with high caries risk, as clinically indicated. Chlorhexidine may be used short- term for antimicrobial biofilm control, but is undesirable long-term due to taste alteration and staining and should be used in a targeted manner [25]. Management includes additional lubrication with gels and foams of artificial saliva, denture hygiene and periodic adjustments that may improve comfort and reduce trauma. However, prosthetic management may be more complex due to reduced saliva, which may affect retention or reduced friction, which may create greater denture movement and frictional trauma.

## Emerging and adjunctive therapies:

Nonpharmacologic gland-stimulating modalities such as low-level laser therapy/photobiomodulation, transcutaneous electrical nerve stimulation (TENS), acupuncture and intraoral electrostimulation have shown mixed results and systematic reviews have concluded the results are improving but are not yet strong enough for routine recommendations [26]. Regenerative and gene therapies remain in the research phase and require further study.

## Patient education and follow-up:

Education is a management strategy. Clinicians should explain the cause of the xerostomia and the correct oral hygiene procedures, list possible products such as safe sugar-free salivary substitutes and xylitol gums and danger signs such as rapid caries development, candidiasis and denture problems. The follow-up plan includes reassessing symptoms using XI or VAS, repeating objective flow measurement and systematically following up with medical providers to prescribe systemic therapy or adjust other medications when appropriate. The management of these conditions is chronic and multimodal, with combination therapy being the preferred solution.

## Preventive measures and patient education for dry mouth:

## Overview:

The treatment for dry mouth naturally encompasses both prevention and personal education to ensure effective management of the condition. Since the decrease of saliva secretion leads to various problems in the oral cavity as well as causes a drop in the patient's life quality, medical professionals are able to guide patients on how to keep the mouth moist and how to prevent the occurrence of complications along with the implementation of risk reduction risk strategies, the promotion of adherence to clinical guidelines and the encouragement of behavior modification [27].

## Pharmacological risk assessment and modification:

Generally, patients who are on antihistamines, tricyclic antidepressants, anticholinergics, diuretics and antihypertensives therapy and complain of dry mouth are experiencing the effects of drug-induced blocking of muscarinic receptors or the activation of the sympathetic nervous system. Thus, healthcare providers are expected to examine the medication regimen with the aim of identifying the agents that provoke xerostomia and deciding where safer alternatives such as SSRIs can replace them. Healthcare professionals can also facilitate symptom relief by changing the drug dose or its timing, such as taking medicine together with food [28].

## Hydration and lifestyle modifications:

Correct hydration is a mandatory prerequisite for salivary glands to work well. Patients should drink 2-3 liters of water every day in small, frequent sips and can choose to operate a humidifier while sleeping. In addition to smoking cessation, cutting back on caffeine and alcohol and refraining from breathing through the mouth, these interventions collectively help stimulate saliva flow and keep oral mucosa healthy [29].

## Preventive dental care protocols:

The restricted buffer role of saliva makes dentition more susceptible to caries and oral mucosa abrasive traumas [30]. Patients suffering from this condition should be examined by their dentist every 3-4 months and follow a sugar- and acid-reduced diet. The use of xylitol-containing sugarless gum allows the salivary glands to produce natural saliva and supports enamel remineralization [31]. Chewing sugar-free gum containing xylitol has been shown to significantly reduce caries risk. The nutrition advisor should encourage the consumption of water-rich fruits and vegetables while warning against abrasive and sticky foods [30].

## Patient education strategies:

## Comprehensive self-care programs:

Well-designed self-care programs elevate the level of patient involvement and commitment. The learning process should encompass the right way of brushing, interdental cleaning, application of high-fluoride dental products and arranging regular dental visits, along with guidance on correct hydration, humidifier use and behavioral modifications [32].

## Saliva substitutes:

Relief provided by sprays, gels and lozenges is relatively short (30 minutes-2 hours). Patients should understand the differences between mucin- and carboxymethylcellulose-based products and choose cost-effective options. Applying substitutes before meals or at bedtime improves comfort during talking, eating or swallowing [33].

## Denture and oral appliance management:

One of the best ways to take care of dentures is by moisturizing them with water before insertion and applying hydrating gels to reduce friction and prevent sores, especially valuable for complete denture wearers managing xerostomia-related complications [27].

## Multidisciplinary coordination and support:

Systemic conditions such as Sjögren's syndrome require collaborative care involving rheumatologists, endocrinologists and oral health professionals [32]. Patient education should emphasize that xerostomia is usually a long- term condition requiring continuous preventive care and early management of complications, while connecting patients with peer support networks and mental health resources can improve emotional adjustment and quality of life [33].

## Conclusion:

Xerostomia is a condition characterized by insufficient saliva production, leading to a dry mouth. It commonly results from medication side effects, autoimmune diseases like Sjögren's syndrome, radiation therapy and other health conditions. This condition increases the risk of dental complications, affects speech and swallowing and requires patient specific treatment, including symptomatic management for dental care and addressing underlying causes to improve quality of life.
